# Medical Expenditure for Chronic Diseases in Mexico: The Case of Selected Diagnoses Treated by the Largest Care Providers

**DOI:** 10.1371/journal.pone.0145177

**Published:** 2016-01-08

**Authors:** Alejandro Figueroa-Lara, Miguel Angel Gonzalez-Block, Jose Alarcon-Irigoyen

**Affiliations:** 1 Division of Technology Management and Innovation, Mexican Social Security Institute, Mexico City, Mexico; 2 Escuela Militar de Graduados de Sanidad, Mexico City, Mexico; 3 Universidad Anáhuac, Mexico City, Mexico; 4 Health Policy and Program Design, PwC, Mexico City, Mexico; 5 PwC, Mexico City, Mexico; London School of Hygiene and Tropical Medicine, UNITED KINGDOM

## Abstract

**Background:**

Chronic diseases (CD) are a public health emergency in Mexico. Despite concern regarding the financial burden of CDs in the country, economic studies have focused only on diabetes, hypertension, and cancer. Furthermore, these estimated financial burdens were based on hypothetical epidemiology models or ideal healthcare scenarios. The present study estimates the annual expenditure per patient and the financial burden for the nine most prevalent CDs, excluding cancer, for each of the two largest public health providers in the country: the Ministry of Health (MoH) and the Mexican Institute of Social Security (IMSS).

**Methods:**

Using the Mexican National Health and Nutrition Survey 2012 (ENSANUT) as the main source of data, health services consumption related to CDs was obtained from patient reports. Unit costs for each provided health service (e.g. consultation, drugs, hospitalization) were obtained from official reports. Prevalence data was obtained from the published literature. Annual expenditure due to health services consumption was calculated by multiplying the quantity of services consumed by the unit cost of each health service.

**Results:**

The most expensive CD in both health institutions was chronic kidney disease (CKD), with an annual unit cost for MoH per patient of US$ 8,966 while for IMSS the expenditure was US$ 9,091. Four CDs (CKD, arterial hypertension, type 2 diabetes, and chronic ischemic heart disease) accounted for 88% of the total CDs financial burden (US$ 1.42 billion) in MoH and 85% (US$ 3.96 billion) in IMSS. The financial burden of the nine CDs analyzed represents 8% and 25% of the total annual MoH and IMSS health expenditure, respectively.

**Conclusions/Significance:**

The financial burden from the nine most prevalent CDs, excluding cancer, is already high in Mexico. This finding by itself argues for the need to improve health promotion and disease detection, diagnosis, and treatment to ensure CD primary and secondary prevention. If the status quo remains, the financial burden could be higher.

## Introduction

Chronic diseases (CDs) are currently the primary cause of death worldwide, leading to more deaths annually than all other causes combined [1]. In 2008, approximately 63% (i.e. 36 million of 57 million) of deaths worldwide resulted from CDs, of which 48% (17 million) and 21% (7.6 million) were due to cardiovascular diseases and cancer, respectively [1]. Furthermore, CDs account for 54% of the global disability-adjusted life years [2]. The United Nations Secretary General Ban Ki-Moon described CDs as a “public health emergency” [3]. According to the World Economic Forum, each 10% rise in CD prevalence is associated with 0.5% lower rates of annual economic growth [4]; thus, CDs have been identified as a global risk and threat to economic development [1]. Additionally, the World Health Organization estimates that CD deaths will increase by 15% globally between 2010 and 2020 [1].

Around 45.6 million CD deaths occur annually in low- and middle-income countries [1]. In Mexico, CDs represent the greatest challenge for the national health system [5]. Indeed, CDs led to 437,800 deaths in a population of 111.2 million in 2008, and accounted for 71% of the total disability-adjusted life years in 2010 [1,6,7]. Between 2000 and 2012, the prevalence of diabetes increased by approximately 60%, from 5.7% to 9.1%, among adults aged 20 years or older, placing diabetes as the first cause of death [8]. In the same period and population, the prevalence of hypertension increased from 30.1% to 31.5% [9]. Breast and prostate cancer are also high priority public health problems in Mexico [10,11]; breast cancer has been the second cause of death among women aged 30–54 years since 2006 [10], while prostate cancer is the most deadly malignancy among Mexican men [12]. Nevertheless, despite the large burden, access to CD services and effective coverage of interventions in Mexico is reportedly low. For example, the proportion of undiagnosed diabetics represents between 18% and 26% of the total population affected by the disease [13,14]. Among diagnosed diabetics, only 78% have at least two medical consultations per year [15]. Further, compliance with national diabetes control guidelines is low, with only 52.7% of diabetics obtaining a blood glucose test, 14.6% having their feet checked, and 9.6% obtaining an HbA1c test at the time of a regular physician visit [15].

The Mexican health system is comprised of public and private institutions. Public institutions are composed of several social security institutes which provide medical health services to persons in the formal economy, the largest of which is the Mexican Institute of Social Security (IMSS). Other public institutions provide health services to persons in the informal economy and the self-employed, the most important being the Ministry of Health (MoH). Private institutions provide healthcare to persons with a capacity to pay, although most Mexicans purchase some health care out-of-pocket [16]. In 2013, out-of-pocket expenditure contributed to 44% of the total health expenditure in Mexico [17].

MoH provides a comprehensive health services package of 285 outpatient, general hospital, and specialized interventions, including drugs and laboratory and other tests; most of this package is financed from general taxation [18]. Of the total MoH outpatient beneficiaries, 83% reported being satisfied with the quality of the medical services received [19]. IMSS does not have an explicit health service package and provides, in theory, treatment for all health needs except for aesthetic interventions, including drugs, laboratory and other tests, and prostheses. Beneficiaries also have access to a wide range of social, cultural, and economic benefits. IMSS health services are financed by employer and employee fees, the government, and IMSS self-accrued income from investments, contributing 66%, 31%, and 3% of the total, respectively [20,21]. Up to 77% of IMSS outpatient care users report being satisfied with the quality of the medical services provided [19]. All services included in the MoH benefit package and all care provided by IMSS are free of charge at the point of use.

The MoH and IMSS are the most important health institutions in Mexico, jointly serving to 65% of the Mexican population [22]. In 2013, with respect to outpatient medical units, MoH had 68% (14,247) and IMSS 5% (1,141) of the total government health facilities (20,822) and, with regards to hospital medical units, MoH had 55% (734) and IMSS 20% (264) of the total number of hospitals in the public health sector [23]. The 2013 total health expenditure was US$ 17.9 billion dollars for MoH and US$ 16.1 billion for IMSS, jointly comprising 84% of the Mexican government’s health expenditure [17].

Medical expenditure projections for selected CDs, such as diabetes [24,25], hypertension [26], breast cancer [27], and asthma [28], posit a crisis scenario. Despite concerns regarding the economic burden of CDs in Mexico, only a handful of medical care expenditure estimates have been produced, focusing mostly on diabetes, hypertension, and breast cancer [24–27]. Furthermore, these estimates are based on hypothetical epidemiologic models and assume compliance with treatment guidelines. To redress this situation, the present study aims to estimate the annual medical care expenditure incurred by the MoH and IMSS, for the most prevalent CDs in Mexico, based on the best evidence available on actual utilization rates and costs for each CD.

## Methods

This is a cross-sectional economic study undertaken from the payer perspective. Expenditure incurred by each of MoH and IMSS in catering for the services demanded by the populations is estimated. Nevertheless, expenditure incurred by IMSS affiliates or MoH beneficiaries when using private or other providers is not included in the analysis.

### ENSANUT 2012 characteristics

The Mexican National Health and Nutrition Survey (ENSANUT) 2012, a nationally representative survey, was the main source of data. ENSANUT was undertaken in the second semester of 2011 and the first of 2012 and surveyed 194,900 persons, representative of 114.9 million persons. The data used was obtained from the Household Members (HM), Health Services Utilization (HSU), and Adult Health sections of the survey. ENSANUT obtained ethical approval from the National Institute of Public Health’s Ethics Committee [29].

### Definition of the analytical sample

The study included only adults (≥20 years old) that reported being beneficiaries of MoH or IMSS, given that these were the only two institutions with a sufficient sample size to estimate expenditure from specific CDs. The study identified the specific CD reported by patients based on the HSU section question, “In the last year, did a doctor diagnose you with any of the following chronic diseases?” The study excluded patients reporting more than one CD, given that ENSANUT did not ask for multiple motives of consultation of hospitalization. Hence, the exclusion avoids inflating expenditure for multi-chronic health care. A total of 20% of the sample reported more than one CD and was thus excluded.

**Fig 1:**
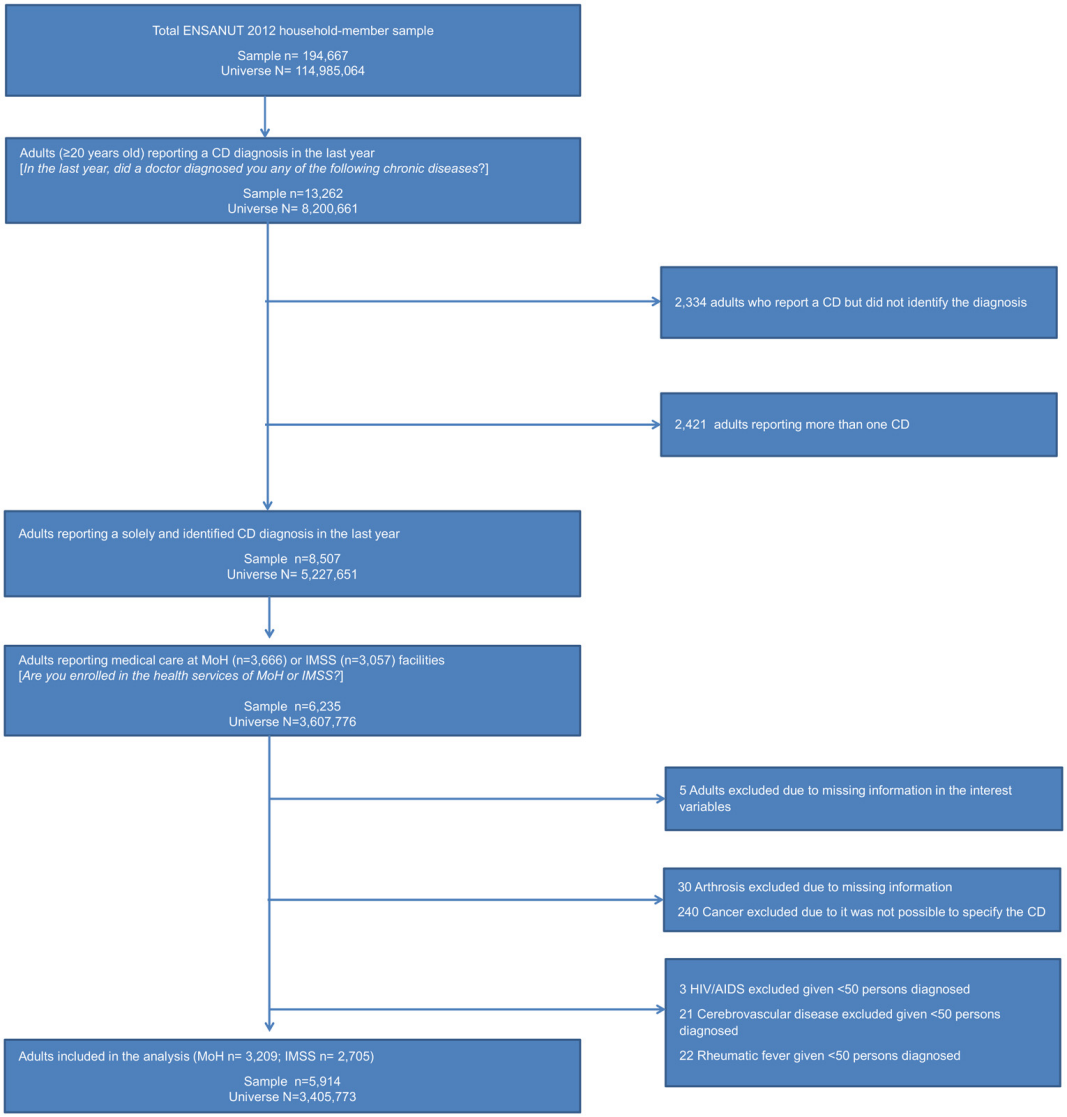
Filters applied to the original data set to obtain the analysis sample.

Cases were excluded in a number of conditions. Arthrosis cases were excluded given that ENSANUT failed to be included as an outpatient utilization motive in the HSU section. All cancer cases had to be excluded given that ENSANUT did not ask patients to report the specific diagnosis. HIV-AIDS, cerebrovascular diseases, and rheumatic fever were excluded due to the insufficient number of cases (less than 50). Fig 1 shows the process used to define the analytical sample according to the structure of questions in ENSANUT.

### Specification of CD diagnosis

Respondents were asked to specify the CD diagnosis from a list of generic disease names through the HM section question, “In the last year, did a doctor diagnose you with any of the following chronic diseases?” The researcher then proceeded to read a list of 16 generic disease names, of which the respondent could confirm up to three. The diagnoses reported by ENSANUT were modified to conform to the most prevalent diseases within the family of diseases of the International Classification Diseases 10^th^ version [30]. This correction aimed to reduce the bias inherent in the estimation of expenditures for disease families. The correction was based on the best evidence available regarding the most frequent specific diseases within the family [31–39]. CDs with important corrections were “diabetes”, categorized as type 2 diabetes mellitus (E11), “colitis” as irritable bowel syndrome (K58), “renal disease” as chronic kidney disease (CKD; N18), “arthritis” as osteoarthritis (M15-M19), and “heart disease” as chronic ischemic heart disease (CIHD; I25).

### Estimation of health services consumption

#### Outpatient health care

Outpatient health care is defined in ENSANUT as any medical consultation that did not require hospitalization and was provided by a general physician or a specialist. Outpatient care also included laboratory and other tests (such as imaging) and drugs prescribed. ENSANUT did not include questions regarding kidney dialysis.

ENSANUT questioned the need for medical consultations in the past two weeks in the HM section through the question, “In the past two weeks, did you receive a consultation due to diseases, disease control, lesions, or accident?” If positive, the respondent was subjected to random sampling to respond to the HSU section. Up to 80% of respondents with CDs were sampled. In the HSU section, respondents were asked to declare the specific motive of medical outpatient consultation through the question, “In the past two weeks, the main reason for medical consultation was…” Respondents freely stated the main consultation motive, which was then codified according to a list of 45 possible responses. Data for respondents that did not state one of the nine CD diseases analyzed was excluded from the analysis. Furthermore, data was also excluded if the CD diagnosis reported in the HM section did not coincide with the CD reported as the motive of utilization.

The average number of medical consultations per patient per year for each CD was calculated by estimating the probability of health service utilization for persons reporting each specific CD. The number of medical consultations per year for each CD were imputed by dividing the total number of persons reporting a specific CD diagnoses in the last year by the total number of persons who utilized health services in the past two weeks. Given that only a sample of respondents was selected to provide information on service utilization in the HSU section, the probability of selection was calculated for each CD and used to weigh the imputed figure. These probabilities were then multiplied by the number of fortnights in the year (26.1).

The average proportion of medical consultations provided by general physicians and specialists for each CD were obtained from the question, in the HSU section, “Who provided care when you attended the consultation?” Respondents reported whether laboratory and other tests were ordered and the number of drugs prescribed as part of a medical consultation in the last two weeks. This information was obtained from the HSU section through the question, “In the medical consultation, did the physician ask you to have any lab or other tests (for example, blood or urine, x-rays, ultrasound, electrocardiogram)?” Information for drugs was obtained from the HSU section through the question, “In the medical consultation, how many drugs did the physician prescribe you?”

However, ENSANUT does not provide information regarding the kind or number of laboratory and other tests or the specific drug or dosage prescribed. To fill these gaps, this information was estimated by an expert panel based on the CD diagnosis reported by the beneficiaries for the consultation in question. The expert panel was asked to provide the missing information based on the Mexican clinical practice guidelines for each CD presented [40]. Therefore, it was assumed that the health care consumption for laboratory and other tests and drugs followed the clinical guidelines. The expert panel consisted of two independent general physicians. When in disagreement, another general physician was asked to provide a third opinion. To ascertain the drugs prescribed, the expert panel was asked to name up to three drugs, in the order established by clinical guidelines. Thus, the type and average number of laboratory and other tests as well as the type, average number, and average dosage of drugs for each CD were identified. The information provided by the expert panel was assumed to be the same for MoH and IMSS patients given that guidelines were developed by a joint institutional committee [40]. Drug consumption estimations assumed that all drugs prescribed were filled, supported by the fact that IMSS beneficiaries obtain their drugs from the provider in 95% of cases [21], while MoH beneficiaries do so in 90% of cases [41].

Given that ENSANUT does not include the consumption of kidney dialysis, this was estimated for beneficiaries who reported a CKD diagnosis and motive of utilization. The consumption was calculated using information reported in the scientific literature; the number of kidney dialysis sessions was differentiated in the literature for each of MoH [42] and IMSS [43]; the proportion of peritoneal dialysis and hemodialysis treatments received was estimated for the sample as a whole. The type of kidney dialysis was randomly imputed to the beneficiaries based on the proportion of patients expected in each kidney dialysis scheme [44,45]. The prevalence of the number of persons in need of kidney dialysis was assumed to be the same for MoH and IMSS.

#### Hospitalization

Hospital care was analyzed according to the number of hospitalization days and the use of general ward and intensive care unit (ICU) beds. The use of hospital care was observed from the question in the HM section, “In the last year, were you hospitalized or admitted?” Beneficiaries who reported hospitalization due to illness or surgery were included in the calculation of the number of total bed-days. The hospitalization cause was observed through the question of the HM section, “Why were you hospitalized in the last year?” The number of total bed-days was observed from the HM section question, “In total, how many days were you hospitalized in the last year?” Hospitalizations with an outlier number of bed-days (fourteen cases) were assigned a maximum of 30 bed-days in order to avoid biasing the health expenditure mean for each CD by accounting days that could have responded to other reasons besides the CD in question.

Given that ENSANUT does not differentiate between bed-days spent in general ward and those in ICU, this information was obtained from estimations based on Mexican data for Diagnostic-Related Groups [46] for each CD, except for hypertension and type 2 diabetes, for which the scientific literature was consulted [47]. This ratio was assumed to be the same for MoH and IMSS.

### Cost of health services

For MoH, the costing sources listed below were used. General physician and specialist consultation costs were observed from the Universal Catalogue of Health Services (CAUSES for its acronym in Spanish), a financial tool widely used by the national MoH payer to fund local government health service providers [48,49]. The costs for laboratory and other tests were obtained from the fee schedule of a representative local health service provider [49]. Prices for drugs were obtained from the 2012 mandatory federal purchasing guidelines [50]. The costs for dialysis and hemodialysis sessions were obtained from activity-based costing published in the scientific literature [43,49]. The cost for ICU bed-days was obtained from the CAUSES catalogue [48]. The general ward bed-day cost was obtained from the top-tier fee schedule of a representative MoH general hospital in Mexico City [51]; the top-tier is representative of health services production costs [52].

For IMSS, the costing sources detailed below were used. The official fee schedule for services rendered to non-beneficiaries was employed for general and specialist physician consultations, laboratory and other tests, and bed-days in general ward and ICU [53]. Published purchase reports were used to obtain costs for drugs [54]. As with the MoH, dialysis and hemodialysis costs were obtained from the published literature for IMSS-specific activity-based costing [43,55].

All costs are presented in current US dollars for 2014, adjusted using the consumer price index as necessary and undertaking a foreign currency conversion using annual exchange rates published by the International Monetary Fund [56,57].

### Statistical analysis

Annual health services consumption expenditure per CD was calculated by multiplying the quantity of services consumed by the unit cost of each health service. The annual total expenditure per patient per CD was obtained by adding the expenditure from medical consultations, laboratory and other tests, prescribed drugs, and hospitalization days. For CKD, the cost of kidney dialysis was also included. The 95% confidence interval for the total annual expenditure per CD and per expenditure component was calculated using the bootstrap technique based on 500 resampling exercises. Herein, confidence intervals reflect only the variation in health care consumption and do not reflect cost vectors. A bivariate statistical analysis using Wald test was performed to explore the difference in health services consumption between MoH and IMSS. All data were analyzed in Stata 10.1 statistical software.

### Estimation of the financial burden due to CD for MoH and IMSS

To estimate the financial burden due to the CDs analyzed, the national prevalence of each CD in adults was first identified. As stated above, ENSANUT identifies the beneficiaries with a CD diagnosis by assessing CD diagnoses in the past year. In order to strengthen the inference of current prevalence, the present study obtained CD prevalence from the published literature for all but depressive disorder and CIHD [9,34,58–62], for which ENSANUT enabled an accurate estimation by asking, in the Adult section, “Have you ever been told by a doctor that you have depression?” and “Have you ever been told by a doctor that you have a chronic heart disease?”

In order to estimate the total number of MoH and IMSS adult beneficiaries with a CD, the total MoH and IMSS adult affiliates was first estimated based on official 2012 affiliation reports [6,22]. The prevalence per CD was then multiplied by the total adult affiliates. In order to estimate the total number of adult CD beneficiaries who consumed outpatient health services per year, the number of beneficiaries with a CD were multiplied by the percentage of beneficiaries who consumed health services according to the published literature [63–69]. In this case, ENSANUT could not be used given that consumption data is only available for those beneficiaries declaring having used services in the last two weeks. Thus, it was not possible to infer which patients had not used services at all in the past year. In the case of inpatient care, annual consumption was inferred from ENSANUT given that hospitalization data was obtained for the past year. Finally, to obtain the financial burden for each institution per CD and per expenditure component, the number of beneficiaries with a CD who had consumed health services in the past year was multiplied by the estimated expenditure per CD. The CD financial burden was assessed against the total annual institutional health expenditure per institution and as a percentage of the gross domestic product (GDP) [17].

## Results

### Analytical sample and beneficiaries diagnosed with a CD

Overall, 7% (14,270) of those surveyed in ENSANUT declared a physician-diagnosed CD. The resulting sample used to estimate the actual expenditure due to medical care for the selected nine CDs was composed of 5,914 beneficiaries, representing 3.4 million beneficiaries and 45% of the total CD diagnoses at the national level. A total of 3,209 MoH beneficiaries and 2,705 IMSS beneficiaries reported a CD (Fig 1). For both health institutions, arterial hypertension was the most frequently reported CD, followed by gastritis, type 2 diabetes, and irritable bowel syndrome; these three diseases represent 81% of the reported CDs for both health institutions.

### Outpatient health services consumption

The use of medical consultations was higher in IMSS beneficiaries, with 12% reporting a consultation in the last two weeks, compared to 10% of MoH beneficiaries (Table 1). CIHD was the only CD where the use of consultations was statistically different across the two health institutions (*F* <0.05). Beneficiaries in MoH who reported having been diagnosed with CKD (14%) or asthma (13%) had the highest percentage of utilization. In the case of IMSS, beneficiaries with CIHD (17%) or CKD (16%) had the highest percentage of utilization.

**Table 1:** Number of insured reporting in ENSANUT a chronic diseases diagnose and utilization of ambulatory health service, by health institution.

Chronic disease	Number of persons reporting a CD diagnosis in the last year (%)				% of persons reporting consultations in the last two weeks		Number of medical consultation per person per year, due to CD		% of consultation provided by a general physician		% of consultation provided by a specialist		% of persons who were prescribed laboratory or other tests		% of persons who were prescribed drugs	
	MoH	%	IMSS	%	MoH	IMSS	MoH	IMSS	MoH	IMSS	MoH	IMSS	MoH	IMSS	MoH	IMSS
Arterial hypertension	1,042	32%	918	34%	12%	10%	3.1	2.7	95%	93%	5%	7%	26%	37%	92%	93%
Gastritis	710	22%	519	19%	7%	8%	1.8	2.1	97%	91%	3%	9%	25%	36%	97%	95%
Type 2 diabetes	486	15%	436	16%	11%	13%	2.9	3.4	97%	87%	3%	13%	45%	55%	92%	92%
Irritable bowel syndrome	376	12%	306	11%	7%	9%	1.8	2.4	82%	86%	18%	14%	41%	19%	94%	95%
Depressive disorder	139	4%	133	5%	9%	12%	2.3	3.2	44%	67%	56%	33%	22%	17%	89%	92%
Chronic kidney disease	130	4%	101	4%	14%	16%	3.7	4.2	86%*	23%	14%*	77%	50%*	69%	86%	100%
Osteoarthritis	114	4%	105	4%	9%	13%	2.4	3.4	63%	73%	37%	27%	25%*	64%	100%	82%
Chronic ischemic heart disease	107	3%	115	4%	7%*	17%	1.7*	4.4	33%*	41%	67%	59%	50%	53%	100%	94%
Asthma	105	3%	72	3%	13%	13%	3.5	3.4	100%	67%	0%	33%	31%	67%	92%	83%
Total/Average	3,209	100%	2,705	100%	10%	12%	2.6	3.2	77%	70%	23%	30%	35%	46%	94%	92%

Source: Own processing based on ENSANUT 2012 information.

Notes: CD = Chronic disease; MoH = Ministry of Health; IMSS = Mexican Institute of Social Security.

*F<0.*05*.

With regards to the intensity of use of medical consultations, IMSS beneficiaries had 3.2 consultations per person per year, whereas MoH beneficiaries had 2.6, although the difference was not significant. For MoH, the highest number of consultations per person per year was for beneficiaries with CKD (3.7) or asthma (3.5). In the case of IMSS, beneficiaries with CIHD (4.4) or CKD (4.4), had the highest number of consultations. The intensity of use of medical consultations between the two health institutions was statistically different across beneficiaries with CIHD (*F* <0.05).

Regarding the medical consultations provider, MoH beneficiaries received more consultations from a general physician (77%) compared to IMSS beneficiaries (70%). The percentage of beneficiaries treated by the general physician was significantly different between health institutions for beneficiaries with CKD, and CIHD (*F* <0.05). MoH beneficiaries diagnosed with asthma reported that 100% of the consultations were provided by a general physician; in contrast, beneficiaries diagnosed with CIHD reported that 67% of the consultations were provided by a specialist. Among IMSS beneficiaries, those diagnosed with arterial hypertension declared the highest percentage of consultations provided by a general physician (93%), while those diagnosed with CKD received the highest percentage of consultations by a specialist (77%).

In general, IMSS prescribed laboratory and other tests to a higher percentage of beneficiaries (46%) compared to MoH (35%), with the percentage being significantly different between health institutions for beneficiaries with CKD and osteoarthritis (*F* <0.05). Among MoH beneficiaries, 50% and 22% of those with CKD, CIHD and depressive disorder, respectively, were prescribed laboratory and other tests. In the case of IMSS beneficiaries, 69% and 17% of those diagnosed with CKD and depressive disorder were ordered laboratory and other tests, respectively.

The drug prescription rates were 94% and 92% for MoH and IMSS beneficiaries, respectively, no difference being statically significant was found. All MoH beneficiaries diagnosed with osteoarthritis or CIHD, while only 86% of those diagnosed with CKD, were prescribed drugs. On the other hand, all IMSS beneficiaries diagnosed with CKD were prescribed drugs, whereas the lowest prescription rate (82%) was reported for those diagnosed with osteoarthritis.

### Hospitalization consumption

Regarding hospitalization rate, 8% and 11% of MoH and IMSS beneficiaries, respectively, had been hospitalized in the past year. Hospitalization rates were significantly different between health institutions for beneficiaries with CKD (*F* <0.05). MoH beneficiaries diagnosed with CIHD had the highest hospitalization rate (18%), whereas those diagnosed with irritable bowel syndrome had the lowest (3%). Among IMSS beneficiaries, the highest hospitalization rate was observed for patients diagnosed with CKD (35%), while the lowest for those diagnosed with osteoarthritis (4%). The median length of stay for MoH and IMSS beneficiaries was 3 days, for which 88% of bed-days were spent in general ward and 12% in ICU (Table 2).

**Table 2:** Reported hospitalization by insured diagnosed with a chronic disease in ENSANUT, by health institution.

Chronic disease	% hospitalized in the last year*		Median length of stay in the last year(min-max)*		% of hospitalization in general ward**	Information source for hospitalization ward
	MoH	IMSS	MoH	IMSS	MoH and IMSS***	
Arterial hypertension	5%	6%	3 (1–30)	3 (1–30)	60%	[47]
Gastritis	5%	5%	3 (1–15)	2 (1–25)	100%	[77]
Type 2 diabetes	6%	7%	3 (1–30)	3 (1–30)	71%	[47]
Irritable bowel syndrome	3%	5%	1 (1–6)	2 (1–30)	100%	[46]
Depressive disorder	6%	6%	2 (1–8)	11 (1–30)	100%	[46]
Chronic kidney disease	16%	35%	3 (1–30)	4 (1–30)	100%	[46]
Osteoarthritis	6%	4%	3 (1–4)	8.5 (4–30)	100%	[46]
Chronic ischemic heart disease	18%	24%	4 (1–15)	7 (1–22)	65%	[46]
Asthma	9%	8%	3 (1–30)	1.5 (1–15)	100%	[46]
Average/median	8%	11%	3 (1–30)	3 (1–30)	88%	

Source:

*Own processing based on information of ENSANUT 2012;

**Other information source.

Notes:

*** = the remaining percentage of hospitalization refers to stay in the intensive care unit; MoH = Ministry of Health; IMSS = Mexican Institute of Social.

### Estimation of the annual expenditure per CD diagnosis

In relation to the estimated annual expenditure per CD diagnosis and expenditure category for both health institutions (Table 3), the four most expensive CDs were CKD, CIHD, type 2 diabetes, and arterial hypertension. The total annual expenditure for CKD was US$ 8,966 and US$ 9,091 for MoH and IMSS, respectively, whereas for CIHD, arterial hypertension and type 2 diabetes the annual expenditure was US$ 440, US$ 198, and US$ 184 for MoH and US$ 2,800, US$ 598, and US$ 691 for IMSS, respectively (Table 3).

**Table 3:** Average annual health expenditure and confidence interval (95%) per person with any expenditure per expenditure component within each health institution (USD for 2014).

	Total expenditure(A) + (D) + (E) + (F)		Medical consultation(A) = (B) + (C)		General physician consultations (B)		Specialist consultations(C)		Laboratory and other tests(D)		Drugs(E)		Hospitalization(F) = (G) + (H)		General ward hospitalization (G)		ICU(H)	
Chronic disease/Health institution	MoH	IMSS	MoH	IMSS	MoH	IMSS	MoH	IMSS	MoH	IMSS	MoH	IMSS	MoH	IMSS	MoH	IMSS	MoH	IMSS
Arterial hypertension	198 (129–268)	598 (409–786)	28 (28–28)	121 (121–121)	26 (26–26)	106 (106–106)	2 (2–2)	15 (15–15)	5 (3–7)	3 (2–4)	1 (1–1)	2 (1–2)	164 (98–230)	472 (279–664)	35 (21–50)	110 (64–155)	129 (77–181)	362 (218–506)
Gastritis	69 (44–91)	198 (131–265)	16 (16–16)	96 (96–96)	15 (15–15)	81 (81–81)	1 (1–1)	15 (15–15)	2 (1–3)	2 (1–3)	1 (1–1)	1 (1–1)	50 (26–75)	99 (33–166)	50 (26–75)	99 (33–166)	0 (0–0)	0 (0–0)
Type 2 diabetes	184 (106–261)	691 (447–934)	26 (26–26)	160 (160–160)	25 (25–25)	125 (125–125)	1 (1–1)	35 (35–35)	12 (6–18)	7 (4–10)	1 (1–1)	1 (1–1)	145 (60–230)	522 (247–798)	45 (20–69)	173 (87–259)	100 (46–155)	349 (172–526)
Irritable bowel syndrome	31 (23–40)	247 (138–357)	17 (17–17)	114 (114–114)	13 (13–13)	87 (87–87)	4 (4–4)	27 (27–27)	2 (1–4)	1 (1–3)	1 (1–1)	1 (1–1)	11 (3–20)	131 (26–236)	11 (3–20)	131 (26–236)	0 (0–0)	0 (0–0)
Depressive disorder	81 (42–118)	570 (252–886)	26 (26–26)	176 (176–176)	9 (9–9)	91 (91–91)	17 (17–17)	85 (85–85)	1 (1–3)	1 (1–1)	4 (1–6)	5 (2–8)	50 (13–86)	388 (70–707)	50 (13–86)	388 (70–707)	0 (0–0)	0 (0–0)
Chronic kidney disease*	8,966 (8,756–9,174)	9,091 (8,368–9812)	35 (35–35)	301 (301–301)	28 (28–28)	41 (41–41)	7 (7–7)	260 (260–260)	19 (5–33)	19 (7–30)	1 (1–1)	1 (1–1)	365 (162–568)	1630 (888–2,373)	365 (162–568)	1,630 (888–2,373)	0 (0–0)	0 (0–0)
Osteoarthritis	103 (51–206)	354 (56–652)	24 (24–24)	110 (110–110)	13 (13–13)	65 (65–65)	11 (11–11)	45 (45–45)	2 (1–4)	7 (2–12)	2 (1–3)	2 (1–3)	75 (27–177)	235 (46–515)	75 (27–177)	235 (46–515)	0 (0–0)	0 (0–0)
Chronic ischemic heart disease	440 (211–668)	2,800 (1756–3847)	20 (20–20)	284 (284–284)	5 (5–5)	76 (76–76)	15 (15–15)	208 (208–208)	6 (1–13)	14 (5–23)	1 (1–2)	4 (2–6)	413 (207–618)	2498 (1454–3543)	104 (49–159)	682 (408–957)	309 (136–482)	1,816 (1,026–2,606)
Asthma	170 (47–294)	430 (149–710)	31 (31–31)	186 (186–186)	31 (31–31)	96 (96–96)	0 (0–0)	90 (90–90)	2 (1–5)	6 (1–11)	4 (2–7)	3 (1–6)	133 (6–260)	235 (53–523)	133 (6–260)	235 (53–523)	0 (0–0)	0 (0–0)

Source: Own processing based on health care consumption identified by the present study.

Notes:

* = includes expenditure of kidney dialysis (dialysis or hemodialysis), for MoH the expenditure was US$ 8,546 and for IMSS was $7,140; CD = Chronic disease; MoH = Ministry of Health; IMSS = Mexican Institute of Social Security; ICU: Intensive Care Unit.

Expenditure for arterial hypertension (the most diagnosed CD) in MoH was due to hospitalization (83%), medical consultation (14%), laboratory and other tests (3%), and drugs (1%). In the case of IMSS, it was due to hospitalization (79%), medical consultation (20%), and laboratory and other tests and drugs (1%, not shown in tables).

For all CDs, annual expenditure was greater for IMSS compared to MoH. The greatest difference was observed for irritable bowel syndrome, with expenditure for IMSS being 7-fold that for MoH. Among the most diagnosed CDs, the estimated expenditure for IMSS for arterial hypertension, gastritis and type 2 diabetes was 2, 1.9 and 2.8 times greater than for MoH, respectively. With respect to expenditure categories, IMSS had a consistently greater expenditure in medical consultations provided by general physicians, specialists, and hospitalizations in general ward and ICU. Regarding drugs, for three (arterial hypertension, depressive disorder and CIHD) of the nine CDs, IMSS had a greater expenditure than MoH, whereas the expenditure level was the same for the remaining CDs, except for asthma where MoH had a greater expenditure than IMSS. For laboratory and other tests, IMSS had a greater expenditure level than MoH in three (osteoarthritis, asthma and CIHD) of the nine CDs (not shown in tables). With respect to kidney dialysis, MoH spent 20% (US$ 8,546) more in comparison with the amount spent (US$ 7,140) by IMSS.

On average, hospitalization represented the greatest annual expenditure (63%), except for CKD, where kidney dialysis contributed to 87% of the expenditure. For both health institutions, CIHD was the CD for which hospitalization represented the greatest percentage of the total expenditure, namely 94% for MoH and 89% for IMSS (not shown in tables).

### Estimation of the actual financial burden due to CD

Considering the prevalence of each of the nine CDs reported and the population covered by MoH and IMSS (Table 4), it was estimated that the MoH was responsible for providing medical care to 36.7 million CD diagnoses among its adult beneficiaries. Thus, during 2012, 14.1 million beneficiaries with one CD diagnosis demanded medical consultations, 4.5 million were prescribed laboratory and other tests, 13.1 million were prescribed medications, 2.2 million were hospitalized, and 75,900 underwent kidney dialysis. In 2012, IMSS was responsible for the medical care of 28.9 million CD diagnoses; of these, 11.1 million demanded medical consultations, 4.7 million were prescribed laboratory and other tests, 10.2 million were prescribed medications 2.2 million were hospitalized and 59,700 underwent kidney dialysis (Table 4).

**Table 4:** Estimated number of chronic disease in insured adults (≥20 year old) and number of insured who utilized health services in the past year, by health institution.

	Adults prevalence (A)	% of adults who utilized consultations in the year (B)	Estimated adults with chronic disease*C = (A) x (adults beneficiaries)		Estimated adults who utilized medical consultationD = (C) x (B)		Estimated adults who utilized laboratory and other tests**E = (D) x (% of beneficiaries who reported use of laboratory and other tests)		Estimated adults who utilized drugs**F = (D) x (% of beneficiaries who reported use of drugs)		Estimated adults who utilized hospitalization in the year***G = (C) x (% of beneficiaries who reported use of hospitalization)		Information source for prevalence and utilization of medical consultations	
Chronic disease	Both health institutions	Both health institutions	MoH	IMSS	MoH	IMSS	MoH	IMSS	MoH	IMSS	MoH	IMSS	Prevalence	Consultations
Arterial hypertension	32%	69%	9,288,968	7,306,804	6,409,388	5,041,695	1,666,441	1,865,427	5,896,637	4,688,776	464,448	438,408	[9]	[63]
Gastritis	31%	13%	9,112,036	7,167,626	1,184,565	931,791	296,141	335,445	1,149,028	885,202	455,602	358,381	[59]	[64]
Type 2 diabetes	9%	87%	2,704,122	2,127,092	2,339,065	1,839,934	1,052,579	1,011,964	2,151,940	1,692,740	162,247	148,896	[60]	ENSANUT
Irritable bowel syndrome	16%	20%	4,718,206	3,711,392	943,641	742,278	386,893	141,033	887,023	705,165	141,546	185,570	[34]	[64]
Depressive disorder	11%	20%	3,161,198	2,486,633	619,595	487,380	136,311	82,855	551,439	448,390	189,672	149,198	ENSANUT	[65]
Chronic kidney disease****	8%	36%	2,359,103	1,855,696	839,841	660,628	419,920	455,833	722,263	660,628	377,456	649,494	[62,67]	[67]
Osteoarthritis	11%	25%	3,096,323	2,435,601	770,984	606,465	192,746	388,137	770,984	497,301	185,779	97,424	[58]	[68]
Chronic ischemic heart disease	3%	40%	807,993	635,576	323,197	254,230	161,599	134,742	323,197	238,977	145,439	152,538	ENSANUT	[69]
Asthma	5%	46%	1,503,928	1,183,006	687,295	540,634	213,062	362,225	632,312	448,726	135,354	94,641	[61]	[66]
Total			36,751,877	28,909,427	14,117,572	11,105,036	4,525,692	4,777,661	13,084,823	10,265,903	2,257,544	2,274,550		

Source: Own processing. Notes: CD = chronic disease; CKD = chronic kidney disease; ENSANUT: Mexican National Health and Nutrition Survey;

* = MoH adults population coverage = 29.4 million; IMSS population coverage = 23.1 million;

** = based on information located in Table 1;

*** = based on information located in Table 2;

**** = 3% of the adults (both health institutions) with CKD are undergoing dialysis (Secretaría de Salud de Mexico (2014) Tratamiento sustitutivo de la función renal. Diálisis y hemodiálisis en la insuficiencia renal crónica. México: SS. 59 p.) For MoH 75,963 insured are under kidney dialysis, in the case of IMSS the insured are 59,753.

The financial burden from CD medical care to MoH and IMSS beneficiaries was US$ 1.42 billion and US$ 3.96 billion, respectively. From the perspective of expenditure categories, in MoH, kidney dialysis represented 45% of the total CD financial burden, whereas 26% was due to medical consultations, 25% to hospitalization, 2% to laboratory and other tests, and 1% to drugs. Regarding IMSS, 48% of the total CD financial burden was due to hospitalization, 40% to medical consultations, 11% to kidney dialysis, and 1% to laboratory and other test and drugs, respectively. CKD, arterial hypertension, type 2 diabetes, and CIHD accounted for most of the CD financial burden of both institutions, amounting to 88% at MoH and 85% at IMSS (Table 5).

**Table 5:** Financial burden of chronic disease (USD for 2014), by health institution.

	Actual financial burden (A) + (B) + (C) + (D)		Annual expenditure due to medical consultation (A) = (consultation expenditure per person per year*) x (adults who utilized medical consultation**)		Annual expenditure due to laboratory and other tests (B) = (laboratory and other tests expenditure per person per year*) x (adults who utilized laboratory and other tests**)		Annual expenditure due to drugs (C) = (drugs expenditure per person per year*) x (adults who utilized drugs**)		Annual expenditure due to hospitalization (D) = (hospitalization expenditure per person per year*) x (adults who utilized hospitalization**)	
Chronic disease/Health institution	MoH	IMSS	MoH	IMSS	MoH	IMSS	MoH	IMSS	MoH	IMSS
Arterial hypertension	269,861,254	831,947,549	179,462,871	610,045,037	8,332,205	5,596,281	5,896,637	9,377,552	76,169,541	206,928,679
Gastritis	43,474,434	126,487,820	18,953,034	89,451,978	592,282	670,890	1,149,028	885,202	22,780,089	35,479,751
Type 2 diabetes	99,124,457	380,889,914	60,815,702	294,389,495	12,630,954	7,083,747	2,151,940	1,692,740	23,525,861	77,723,932
Irritable bowel syndrome	19,259,718	109,775,562	16,041,901	84,619,745	773,786	141,033	887,023	705,165	1,557,008	24,309,620
Depressive disorder	27,935,129	145,992,503	16,109,466	85,778,887	136,311	82,855	2,205,758	2,241,948	9,483,594	57,888,813
Chronic kidney disease***	825,047,618	1,693,484,493	29,394,425	198,848,978	7,978,487	8,660,831	722,263	660,628	137,771,621	1,058,674,662
Osteoarthritis	34,364,539	93,317,333	18,503,625	66,711,117	385,492	2,716,962	1,541,969	994,602	13,933,453	22,894,651
Chronic ischemic heart disease	67,822,917	456,084,207	6,463,943	72,201,426	969,591	1,886,389	323,197	955,906	60,066,186	381,040,485
Asthma	42,263,541	126,317,947	21,306,151	100,557,902	426,123	2,173,348	2,529,246	1,346,178	18,002,021	22,240,519
Total	1,429,153,607	3,964,297,328	367,892,731	1,602,604,566	32,225,231	29,012,336	17,407,061	18,859,921	363,289,375	1,887,181,112

Source: Own processing.

Notes:

* = the expenditure information is located in Table 3;

** = the information about the adults who utilized health services is located in Table 4;

*** = includes de financial burden due to kidney dialysis, for MoH the burden is of USD $649,180,823, for IMSS the burden is of US$ 426,639,394.

The financial burden on the MoH of the nine CDs represented 8% of the total annual MoH health expenditure and 0.12% of the GDP. In the case of IMSS, the CD financial burden represented 25% of the annual IMSS total health expenditure and 0.32% of the GDP. Thus, the total joint CD financial burden for both health institutions was 0.44% of the GDP.

## Discussion

Herein, the expenditure due to medical care of the nine highest prevalence CDs, excluding cancer, cerebrovascular disease, arthrosis and rheumatic fever, has been estimated from the perspective of the main health providers in Mexico–the MoH and IMSS. The present study found that the annual expenditure per CD diagnosis ranged from US$ 31 (irritable bowel syndrome at MoH) to US$ 9,091 (CKD at IMSS). For most CDs, the expenditure driver was hospitalization and, specifically, ICU bed-days, except for CKD, were the driver was kidney dialysis. The expenditure for every CD was greater at IMSS compared to MoH. For both health institutions, the CDs contributing the most to the actual financial burden were CKD, arterial hypertension, type 2 diabetes, and CIHD. The financial burden of the CDs analyzed represents 8% and 25% of the annual MoH and IMSS total health expenditure, respectively, jointly representing 0.44% of the GDP.

The financial burden disparity between MoH and IMSS may be attributed to the unit cost difference for hospitalization and medical consultation, rather than the intensity of health services consumption. Indeed, such differences were not found to be statically significant for most CDs. The general ward and ICU unit cost per bed-day at IMSS was 1.59 and 1.33 times greater, respectively, compared to MoH unit costs, whereas with respect to medical consultations, the cost for a general physician and a specialist at IMSS was 3.8 and 5.2 times greater compared to MoH.

The present study led to significantly different findings than those reported in previous studies. Arredondo and Reyes [26] estimated that the financial burden for the medical care of hypertension for all public providers in 2012 was US$ 3.1 billion, assuming all patients in need received ideal patterns of care. Herein, the total estimated financial burden for MoH and IMSS was US$ 1.1 billion, indicating the importance of analyzing actual data rather than ideal patterns of care.

Arredondo and Reyes [24] also estimated the cost per diabetic patient in 2011 to be US$ 707 if care was provided according to clinical guideline standards, a figure 62% greater than that indicated herein. Mendez et al. [47], using expert panel-based costing estimates, stated an annual per diabetic patient cost of US$ 1,428, a figure 226% greater than the current findings. The differences in the estimates of these two studies further suggest that the approach used herein renders more reliable figures, at least in the case of diabetes. Cortes-Sanabria et al. [55], using a bottom-up costing method, estimated the cost of end-stage renal disease at US$ 14,107, a figure 55% greater than the present finding for CKD in general, thus validating the current approach.

The present study estimated actual expenditure by specific CD diagnoses, as allowed by the currently available data, and in particular that from ENSANUT, a nationally representative survey. Expenditure for specific CDs was established by excluding beneficiaries reporting more than one CD–amounting to 23% of the total surveyed population. Total expenditure for the nine CDs was estimated from national prevalence data, thus taking into consideration the expenditure for multimorbid patients.

Despite its interesting findings, the present study has some important limitations. By relying on CD diagnosis and health services consumption data from the survey, the information used is subject to social desirability [70] and recall biases; both are likely to underestimate the calculated expenditures. Social desirability bias could contribute to a lack of reporting by beneficiaries with a diagnosed CD or to an underestimation of the health services consumption, whereas recall bias could lead to beneficiaries forgetting to report outpatient health services consumed in the past two weeks or hospitalization in the past year. Further, the present study relied on clinical guidelines and expert panel interpretation for the specification of the consumption of laboratory and other tests and drugs, leading to a possible over-estimation of actual provision. Nevertheless, this bias is of minor importance to the overall expenditure estimates given that laboratory and other tests and drugs expenditure represents less than 5% of the total financial burden of both health institutions. Other possible biases include the estimation of the distribution of hospital length of stay between general ward and ICU, of the consumption of kidney dialysis, and of multimorbid CD expenditure. These estimations had to rely on the reported consumption from studies that, whilst reliable, had different purposes and methodologies. However, the kidney dialysis literature was based on costing studies for each of IMSS and MoH [42,43]. Additionally, the total expenditure incurred by multimorbid patients could be higher or lower than the estimates based on single diagnoses. The calculation of expenditure for single CD diagnoses could have biased the financial burden estimation given that the 23% of cases at the national level that are multimorbid could incur a greater expense due to poor health or a lesser expense due to efficiency gains in medical care for more than one disease.

Health service unit costs were based on institution-specific, official, updated cost schedules that are widely applied for economic transactions. However, cost schedules are not necessarily correctly estimated to ensure that institutions charge market or production costs. Indeed, some of the cost schedules used are applied by institutions to charge for services provided to patients not protected by insurance mechanisms or to fund MoH primary care and general hospital expenditures. The cost schedules used herein form the basis of most Mexican health economic studies and have been found to be reliable for diverse purposes [55,71,72].

Another limitation of this study is the assumption that all hospitalizations reported in ENSANUT for surgery and illness were related to the diagnosed CD, with a possible expenditure overestimation. To assess this possible bias, the median length of stay per CD reported by ENSANUT was compared to that per CD reported for each institution in the Automated Hospital Discharge Subsystem [73]. No significant differences were found for most CD across the two databases, suggesting that the assumption was warranted.

## Conclusions

Mexico is undergoing a rapid epidemiological transition, with CDs already occupying the first ranks in morbidity and mortality. Indeed, the financial burden by the two main health institutions of the country reflects this epidemic. The annual expenditure per patient reported herein is not as high as that projected in other studies assuming ideal patterns of care, suggesting that health institutions are facing a large, unmet need due to both undiagnosed illness and under-treatment.

Health institutions should improve health promotion and disease detection, diagnosis, and treatment to ensure primary and secondary prevention. Prevention measures should focus in cost-effectiveness analyses of alternative prevention and treatment pathways to identify the most efficient alternatives and synergistic patterns of health care organization. Integrated health services with the most synergetic arrangements should be sought across diseases with common risk factors and treatment pathways [74]. Costing studies should form an essential component in the design of such interventions [75, 76].

Further research is required to assess the reliability of the unit costs of the main health institutions in Mexico. The present study suggests the need to develop bottom-up costing studies to assess comparability and reliability. National health surveys should be more specific with regard to CD diagnoses. Further studies should also be undertaken to establish the disease stage at diagnosis in order to more accurately ascertain expenditure. With these approaches, costing for cancer should be undertaken. Future studies should also estimate the cost of CDs from the societal perspective in order to achieve a more complete view of the financial burden generated by CDs. Finally, costing studies of multiple CD beneficiaries are also a necessity for stakeholders in Mexico.

## References

[1] World Health Organization (2011) Global status report on noncommunicable diseases 2010. Geneva: WHO 162 p.

[2] Murray C, Vos T, Lozano R, Naghavi M, Flaxman AD, Michaud C et al (2012) Disability-adjusted life years (DALYs) for 291 diseases and injuries in 21 regions, 1990–2010: a systematic analysis for the Global Burden of Disease Study 2010. Lancet 380: 2197–2223. 10.1016/S0140-6736(12)61689-4 23245608

[3] Demaio A, Jamieson J, Horn R, de Courten M, Tellier S. (2013) Non-communicable diseases in emergencies: a call to action. PLoS Curr 5 10.1371/currents.dis.53e08b951d59ff913ab8b9bb51c4d0de PMC3775888 24056956

[4] World Economic Forum (2010) Global risk 2010. Geneva: WEC 52 p

[5] Córdova-Villalobos JA, Barriguete-Meléndez JA, Lara-Esqueda A, Barquera S, Rosas-Peralta M, Hernandez-Avila M et al (2008) Chronic non-communicable diseases in Mexico: epidemiologic synopsis and integral prevention. Salud Publica Mex 50:419–427. 18852939 10.1590/s0036-36342008000500015

[6] Consejo Nacional de Población. Indicadores demográficos de la República Mexicana 1990–2010. Available: http://www.conapo.gob.mx/es/CONAPO/Proyecciones. Accessed 18 September 2014.

[7] Institute for Health Metrics and Evaluation (2013) GBD Compare. Available: http://vizhub.healthdata.org/gbd-compare/. Accessed 11 May 2015.

[8] Juan-López M (2013) El análisis de la ENSANUT 2012 como contribución para las políticas públicas. Salud Publica Mex 55:79–80. 24626716

[9] Campos-Nonato I, Hernández-Barrera L, Rojas-Martínez R, Pedroza-Tobías, Medina-García C, Barquera-Cervera. (2013) Hypertension: prevalence, early diagnosis, control and trends in Mexican adults. Salud Publica Mex 55:144–150. 24626690

[10] Knaul FM, Nigenda G, Lozano R, Arreola-Ornelas H, Langer A, Frenk J. (2009) Breast cancer in Mexico: an urgent priority. Salud Publica Mex 51:335–344. 10.1590/s0036-36342009000800026 19967291

[11] Sánchez-Barriga J (2013) Tendencias de mortalidad y años potenciales de vida perdidos por cáncer de próstata en los 32 estados y en las 7 regiones socioeconómicas de México en el periodo 2000–2010. Gaceta Méd Mex 149:576–585. 24108346

[12] Secretaria de Salud de México (2013). Sistema Nacional de Información en Salud. Principales causas de mortalidad en hombres. Available: http://sinais.salud.gob.mx/mortalidad/. Accessed 25 March 2014.

[13] Vázquez-Martínez JL, Gómez-Dantés H, Fernández-Cantón S (2006) Diabetes mellitus en población adulta del IMSS. Resultados de la Encuesta Nacional de Salud 2000. Rev Med Inst Mex Seguro Soc 44:13–26. 16497255

[14] Escobedo-De la Peña J, Buitrón-Granados LV, Ramírez-Martínez JC, Chavira-Mejía R, Schargrodsky H, Marcet-Champagne B. (2011) Diabetes en México. Estudio CARMELA. Cir Cir 424–431. 22385762

[15] Jiménez-Corona A, Aguilar-Salinas CA, Rojas-Martínez R, Hernandez-Avila M (2013) Diabetes mellitus tipo 2 y frecuencia de acciones para su prevención y control. Salud Publica Mex 55:137–143. 24626689

[16] Gómez-Dantés O, Sesma S, Becerril VM, Knaul FM, Arreola H, Frenk J. (2011) The health system of Mexico. Salud Publica Mex 53:220–232. 21877087

[17] Sistema Nacional de Información en Salud (2015) Boletín de Información Estadística. Available: http://www.dgis.salud.gob.mx/contenidos/publicaciones/p_bie.html. Accessed 11 May 2015.

[18] Secretaría de Salud (2013) Sistema de Protección Social en Salud. México: CNPSS, 169 p.

[19] Reyes-Morales H, Flores-Hernández S, Sauceda-Valenzuela AL, Vértiz-Ramírez JJ, Juárez-Ramírez C, Wirtz V et al (2013) Percepción de los usuarios sobre la calidad de la atención ambulatoria en servicios de salud en México. Salud Publica Mex 55:100–105. 24626684

[20] Diario Oficial de la Federación (2014) Ley del Seguro Social. México: DOF, 126 p.

[21] Instituto Mexicano del Seguro Social (2014) Informe al Ejecutivo Federal y al Congreso de la Unión sobre la situación financiera y los riesgos del Instituto Mexicano del Seguro Social. México: IMSS, 323 p.

[22] Consejo Nacional de Evaluación de la Política de Desarrollo Social (2014) Indicadores de acceso y uso efectivo de los servicios de salud de afiliados al Seguro Popular. Mexico: CONEVAL 89 p.

[23] Secretaria de Salud de México (2013) Sistema Nacional de Información en Salud. Recursos físicos y materiales (infraestructura). Available: http://www.dgis.salud.gob.mx/contenidos/sinais/e_rmateriales.html. Accessed 11 May 2015.

[24] Arredondo A, Reyes G (2013) Health disparities from economic burden of diabetes in middle-income countries: evidence from Mexico. PLoS ONE 7:e68443. 10.1371/journal.pone.0068443 PMC3709919 23874629

[25] Zhang P, Zhang X, Brown J, Vistisen D, Sicree R, Shaw J et al (2010) Global healthcare expenditure on diabetes for 2010 and 2030. Diabetes Res Clin Pract 87:293–301. 10.1016/j.diabres.2010.01.026 20171754

[26] Arredondo A, Zuñiga A (2012) Epidemiological changes and financial consequences of hypertension in Latin America: implications for the health system and patients in Mexico. Cad Saude Publica 28:497–502. 22415182 10.1590/s0102-311x2012000300010

[27] Meneses-García A, Ramírez T, Ruiz-Godoy L, Chiquete E, Mohar A (2012) Costs of breast cancer treatment prior to the introduction of immune-based therapy in Mexico. Rev Med Inst Mex Seguro Soc 50:19–24. 22768813

[28] Tapia-Diaz AM, Casas-Medina GA (2009) Costos de atención y carga de enfermedad de pacientes asmáticos del Instituto Nacional de Enfermedades Respiratorias. Rev Inst Nal Enf Resp Mex 22:182–189.

[29] Romero-Martínez M, Shamah-Levy T, Franco-Nuñez A, Villalpando S, Cuevas-Nasu L, Gutierrez JP et al (2013) National health and nutrition survey: design and coverage. Salud Publica Mex 55:332–340. 24626712

[30] World Health Organization (2015) International classification of Diseases. Available: http://www.who.int/classifications/icd/en/. Accessed 27 May 2015.

[31] Ministry of health of Mexico (2014). National health information system. Number of hospital discharges recorded in the medical units of public institutions in 2011. Available: http://www.sinais.salud.gob.mx/egresoshospitalarios/basesdedatoseh.html. Accessed 22 April 2014.

[32] International Diabetes Federation (2013). Diabetes atlas. Brussels: IDF 159p.

[33] World Health Organization (2012). Fact sheet 132 Diabetes. Geneva: 2012. Available: http://www.who.int/mediacentre/factsheets/fs312/es/. Accessed 22 April 2014.

[34] Lopez-Colombo A, Morgan D, Bravo-Gonzalez D, Montiel-Jarquin A, Mendez-Martinez S, Schmulson M (2012) The epidemiology of functional gastrointestinal disorders in Mexico: a population-based study. Gastroenterol Res Pract 606174:1–8. 10.1155/2012/606174 PMC3313569 22474443

[35] Valerio-Ureña J, Vasquez-Fernandez F, Jimenez-Pineda A, Cortazar-Benitez LF, Azamar-Jácome AA, Duarte-Velázquez ME et al (2010) Prevalencia del síndrome de intestino irritable en población abierta de la ciudad de Veracruz, México. Rev Gastroenterol Mex 71:36–41. 20423781

[36] González-Pier E, Gutierrez-Delgado C, Stevens G, Barraza-Llorens M, Porras-Condey R, Carvalho N et al (2007) Priority setting for health interventions in Mexico´s Systems of Social Protection in Health. Salud Publica Mex 49:S37–S52. 17469398

[37] World Federation for Mental Health (2012) Depression: a global crisis. Occoquan: WFMH 31 p.

[38] Lozano R, Gómez-Dantés H, Garrido-Latorre F, Jiménez-Corona A, Campuzano-Rincón JC, Franco-Marina F et al (2013) Burden of disease, injuries, risk factors and challenges for the health system in Mexico. Salud Publica Mex 55:580–594. 24715011

[39] Ministry of health of Mexico-General Directorate of Epidemiology (2011) Epidemiology in brief. México: MH-DGEPI 7 p.

[40] Secretaría de Salud de México (2014) Catálogo maestro de guías de práctica clínica. Available: http://www.cenetec.salud.gob.mx/interior/catalogoMaestroGPC.html. Accessed 28 April 2014.

[41] Garrido-Latorre F, Hernández-Llamas H, Gómez-Dantés O. (2008) Surtimiento de recetas a los afiliados al Seguro Popular de Salud de México. Salud Publica Mex 50: S429–S436. 19082253 10.1590/s0036-36342008001000003

[42] Afrashtehfar CDM, Pineda-Pérez JA, Afrashtehfar KI (2012) Peritonitis asociada a diálisis peritoneal. Rev Sanid Milit Mex 66:219–224

[43] Duran-Arenas L, Avila-Palomares PD, Zendejas-Villanueva R, Vargas-Ruiz MM, Tirado-Gomez, López-Cervantes M (2011) Costos directos de la hemodiálisis en unidades públicas y privadas. Salud Publica Mex 53 suppl 4: S516–S524. 10.1590/s0036-36342011001000016 22282215

[44] Rosa-Diez G, Gonzalez-Bedat M, Pecoits-Filho R, Marinovich S, Fernandez S, Lugon J et al (2014) Renal replacement therapy in Latin American end-stage renal disease. Clin Kidney J 7:431–436. 25349696 10.1093/ckj/sfu039 PMC4208784

[45] Secretaria de Salud (2014) Tratamiento sustitutivo de la función renal Diálisis y hemodiálisis en la insuficiencia renal crónica. México: SS, 59 p.

[46] Instituto Mexicano del Seguro Social (2011) Grupos relacionados con el diagnóstico. México: IMSS 230 p.

[47] Méndez-Hernández P, Dosamantes-Carrasco D, Siani C, Flores YN, Arredondo A, Lumbreras-Delgado I et al (2011) A workplace physical activity program at a public university in Mexico can reduce medical costs associated with type 2 diabetes and hypertension. Salud Publica Mex 54:20–27. 10.1590/s0036-36342012000100004 22286825

[48] Secretaría de Salud de México (2012) Tabulador del Catálogo Universal de Servicios de Salud 2012–2013. México: CNPSS 5 p.

[49] Gobierno del Estado de Hidalgo (2012) Decreto 369 que aprueban las cuotas y tarifas del organismo descentralizado de la administración pública estatal denominado “Servicios de salud de Hidalgo”, para el ejercicio fiscal 2013. Pachuca: GEH 35 p.

[50] Secretaría de Gobernación de México (2012) Lineamientos para la adquisición de medicamentos asociados al catálogo universal de servicios de salud por las entidades federativas con recursos transferidos por concepto de cuota social y de la aportación solidaria federal del sistema de protección social en salud. México: DOF 31 p.

[51] Secretaría de Hacienda y Crédito Público de México (2013) Tabulador de cuotas de recuperación del Hospital General Dr. Manuel Gea Gonzalez. México: SHCP 23 p.

[52] Secretaría de Salud de México (2005) Manual de procedimientos para la elaboración, presentación, aplicación y operación del tabulador de cuotas de recuperación de servicios médico-asistenciales, así como la asignación de nivel socioeconómico, reclasificación e integración de cuentas corrientes, recepción de pago y reconocimiento de adeudo y cancelación de cuentas incobrables de pacientes. México: SS 160 p.

[53] Secretaría de Gobernación de México (2014) Diario Oficial de la Federación martes 29 de abril 2014. Available: http://www.dof.gob.mx/nota_detalle.php?codigo=5342538&fecha=29/04/2014. Accessed 29 April 2014.

[54] Instituto Mexicano del Seguro Social (2014) Portal de compras del IMSS. Available: http://compras.imss.gob.mx/?P=search_alt. Accessed 18 Jun 2014.

[55] Cortes-Sanabria L, Paredes-Ceseña CA, Herrera-Llamas RM, Cruz-Bueno Y, Soto-Molina-H, Pazarín L et al (2013) Comparison of cost-utility between automated peritoneal dialysis and continous ambulatory peritoneal dialysis. Arch Med Res 44:655–661. 10.1016/j.arcmed.2013.10.017 24211750

[56] International Monetary Fund (2014) Exchange rate archives by month. Available: http://www.imf.org/external/np/fin/data/param_rms_mth.aspx. Accessed 29 April 2014.

[57] Secretaría de Hacienda y Crédito Público de México (2014) Índice nacional de precios al consumidor 2014. Available: http://www.sat.gob.mx/informacion_fiscal/tablas_indicadores/Paginas/inpc_2014.aspx. Accessed 29 April 2014.

[58] Peláez-Ballestas I, Sanin LH, Moreno-Montoya J, Alvarez-Nemegyei J, Burgos-Vargas R, Garza-Elizondo M et al (2011) Epidemiology of the rheumatic diseases in Mexico. A study of 5 regions based on the COPCORD methodology. J Rheumatol Suppl 86:3–8. 10.3899/jrheum.100951 21196592

[59] Herrera-Goepfert R (1999) Chronic gastritis and Helicobacter pylori status in asymptomatic young Mexican volunteers. Rev Inst Nal Cancerol 45(1):24–26.

[60] Hernandez-Ávila M, Gutiérrez JP, Reynoso-Noverón N (2013) Diabetes mellitus in Mexico. Status of the epidemic. Salud Publica Mex 55 suppl 2:129–136. 24626688

[61] García-Sancho, Fernández-Plata R, Martínez-Briseño D, Franco-Marina F, Perez-Padilla JR (2012) Adult asthma in Mexico City. Salud Publica Mex 54:425–432. 22832835 10.1590/s0036-36342012000400013

[62] Amato D, Alvarez-Aguilar C, Castañeda-Limones R, Rodriguez E, Avila-Diaz M, Arreola F et al (2005) Prevalence of chronic kidney disease in an urban Mexican population. Kidney Int Suppl 97:11–17. 10.1111/j.1523-1755.2005.09702.x 16014087

[63] Instituto Nacional de Salud Pública (2012) Encuesta Nacional de Salud y Nutrición 2012 Resultados nacionales. México: INSP 196 p.

[64] Valenzuela J, Alvarado J, Cohen H, Damiao A, Francisconi C, Frugone L et al (2004) Un consenso latinoamericano sobre el síndrome del intestino irritable. Gastroenterol Hepatol 27:325–343. 15117614 10.1016/s0210-5705(03)70470-1

[65] Medina-Mora ME, Borges G, Benjet C, Lara MC, Rojas E, Zambrano J et al (2009) Estudio de los trastornos mentales en México: resultados de la Encuesta Mundial de Salud Mental. Washington: PAHO 89 p.

[66] Neffen H, Gonzalez SN, Fritscher CC, Dovali AE Williams (2010) The burden of unscheduled health care for asthma in Latin America. J Investing Allergol Clin Immunol 20:596–601. 21314001

[67] Cusumano AM, González-Bedat MC (2008) Chronic kidney disease in Latin America: time to improve screening and detection. Clin J Am Soc Nephrol 3:594–600. 10.2215/CJN.03420807 18272831 PMC6631092

[68] Burgos-Vargas R, Cardiel MH, Loyola-Sánchez A, De Abreu MM, Pons-Estel BA, Rossignol M et al (2014) Characterization of knee osteoarthritis in Latin America. A comparative analysis of clinical and health care utilization in Argentina, Brazil, and Mexico. Reumatol Clin 10:152–159. 10.1016/j.reuma.2013.07.013 24286933

[69] Secretaría de Salud (2008) Programa de acción específico 2007–2012 Riesgo cardiovascular. México: SS 76 p.

[70] Levesque JF, Mukherjee S, Grimard D, Boivin A, Mishra S (2013) Measuring the prevalence of chronic diseases using population surveys by pooling self-reported symptoms, diagnosis and treatments: results from the World Health Survey of 2003 for South Asia. Int J Public Health 58:435–447. 10.1007/s00038-013-0446-5 23436012

[71] Ramsey JM, Elizondo-Cano M, Sanchez-Gonzalez G, Peña-Nieves A, Figueroa-Lara A (2014) Opportunity costs for early treatment of chagas disease in Mexico. PLoS Negl Trop Dis 8(4): e2776 10.1371/journal.pntd.0002776 24743112 PMC3990484

[72] Carlos F, Clark P, Maciel H, Tamayo JA (2009) Direct costs of osteoporosis and hip fracture: an analysis for the Mexican Social Insurance Health Care System. Salud Publica Mex 51:108–113. 10.1590/s0036-36342009000700014 19287884

[73] Secretaria de Salud de México (2015) Bases de datos sobre egresos hospitalarios. Available: http://www.dgis.salud.gob.mx/contenidos/basesdedatos/std_egresoshospitalarios.html. Accessed 09 Jun 2015.

[74] Alleyne G, Binagwaho A, Haines A, Jahan S, Nugent R, Rojhani A et al (2013) Embedding non-communicable diseases in the post-2015 development agenda. Lancet 381:566–574. 10.1016/S0140-6736(12)61806-6 23410606

[75] Atun R, de Jongh TE, Secci FV, Ohiri K, Adeyi O, Car J. (2011) Integration of priority population, health and nutrition interventions into health systems: systematic review. BMC Public Health 11:780 10.1186/1471-2458-11-780 21985434 PMC3204262

[76] Bonita R, Magnusson R, Bovet P, Zhao D, Malta DC, Geneau R et al (2013) Country actions to meet UN commitments on non-communicable disease: a stepwise approach. Lancet 381:575–584. 10.1016/S0140-6736(12)61993-X 23410607

[77] Secretaría de salud de México (2011) Profilaxis, diagnóstico y tratamiento de la gastritis aguda (erosiva) en los tres niveles de atención. Mexico: SS 48 p.

